# Microbiota-gut-brain axis imbalance: a promising therapeutic target for preserving brain health in high-altitude environment

**DOI:** 10.3389/fnins.2026.1820153

**Published:** 2026-07-01

**Authors:** Hemiao Xu, Wangyang Chen, Qiming Xiao, Jiahui Ren, Daiyu Yang, Shuai Li, Yanna Cai, Jianyekai Tuerheng, Rou Tang, Kun He, Dong Wu

**Affiliations:** 1 Department of Gastroenterology, State Key Laboratory of Complex Severe and Rare Diseases, Peking Union Medical College Hospital, Chinese Academy of Medical Sciences and Peking Union Medical College, Beijing, China; 2 Department of Gastroenterology, Xizang Autonomous Region People's Hospital, Lhasa, Xizang Autonomous Region, China; 3 Department of Neurosurgery, State Key Laboratory of Complex Severe and Rare Diseases, Peking Union Medical College Hospital, Chinese Academy of Medical Sciences and Peking Union Medical College, Beijing, China; 4 Department of Pharmacy, State Key Laboratory of Complex Severe and Rare Diseases, Peking Union Medical College Hospital, Chinese Academy of Medical Sciences and Peking Union Medical College, Beijing, China

**Keywords:** brain health, gut microbiota, high-altitude, hypoxia, microbiota-gut-brain axis

## Abstract

High-altitude hypobaric hypoxia poses a significant threat to brain function, yet effective neuroprotective strategies remain limited. Emerging evidence highlights the microbiota-gut-brain axis (MGBA) as a key mediator in high-altitude-induced cognitive impairment, positioning it as a potential therapeutic target. This review synthesizes current knowledge on how high-altitude exposure dynamically reshapes gut microbial ecology, characterized by reduced diversity, phylum-level instability, and functional metabolic shifts. Furthermore, we delineate how such altitude-induced dysbiosis has been associated with neural dysfunction through interconnected pathogenic mechanisms that are proposed to link gut ecology to brain outcomes: intestinal barrier disruption with metabolic dysregulation, LPS/TLR4-mediated neuroinflammation, vagal and enteric nervous system alterations, oxidative stress imbalance, and neuroendocrine dysregulation. Most current evidence is correlational, and further research is needed to establish causality. A critical unresolved question is whether short-term, transient gut dysbiosis at high altitude can instigate long-lasting neurological deficits independent of ongoing microbial perturbation. We further evaluate microbiota-targeted neuroprotective strategies, including probiotics, prebiotics, and fecal microbiota transplantation, highlighting their distinct mechanisms and summarizing the current evidence supporting MGBA-targeted interventions for high-altitude brain health. Preclinical studies suggest these approaches hold promise by restoring barrier integrity, attenuating inflammatory signaling, and rebalancing microbial metabolite profiles, while human intervention evidence remains scarce. Finally, we discuss critical challenges and future directions for translating these mechanistic insights into personalized interventions, emphasizing deeper mechanistic exploration and the synergistic interactions among microbial taxa. These insights may inform more effective therapeutic strategies for the growing populations residing in or traveling to high-altitude regions.

## 1 Introduction

High-altitude regions present substantial threats to human health on a global scale, as they are typified by multiple extreme stressors including hypobaric hypoxia, low ambient temperatures, and strong ultraviolet radiation. Currently, more than 80 million individuals reside permanently at elevations above 2,500 meters, while countless others experience temporary high-altitude exposure during travel, occupational activities, or military assignments (Gatterer et al., 2024). Among these environmental stressors, the decrease in oxygen exerts a critical influence on human physiological functions and life activities (Basnyat et al., 1999; Tremblay and Ainslie, 2021). High-altitude hypoxia exerts diverse impacts on the digestive, cardiovascular, and nervous systems, as well as on the metabolism of exogenous compounds, which can cause high-altitude sickness, including acute mountain sickness, chronic polycythemia, cardiovascular disease, pulmonary edema, and cerebral edema (Zhu J. et al., 2022; Chen et al., 2022). As the body’s most oxygen-dependent organ, the brain is particularly vulnerable to hypoxic challenge, with cognitive function undergoing substantial impairment under oxygen deprivation (Kumar et al., 2018). Mounting evidence indicates that high-altitude hypoxia may disrupt multiple cognitive domains, including reduced attention, impaired inhibitory control, lower accuracy in executive function tasks, and prolonged response latencies (Bonnon et al., 1995; Asmaro et al., 2013; Ma et al., 2015; Griva et al., 2017). Despite the well-recognized detrimental effects of high-altitude exposure on brain function, effective strategies for protecting against such impairments in high-altitude regions remain scarce (Zhao et al., 2022).

The bidirectional communication between the gastrointestinal tract and the central nervous system (CNS) has long been recognized under the framework of the gut-brain axis (GBA; Foster et al., 2017). This classical concept encompasses neural, endocrine, and immune pathways that enable continuous crosstalk between the gut and the brain (Che Mohd Nassir et al., 2025). In recent years, this framework has undergone an expansion with the recognition of the gut microbiota as a critical intermediary in gut-brain communication. The emerging concept of the microbiota-gut-brain axis (MGBA) positions the trillions of microorganisms residing in the intestine, collectively known as the gut microbiota, as a central hub that modulates and integrates signals along this axis. MGBA is gaining increasing attention in the basic biological and physiological studies in the field of psychiatry, neurodevelopment, and age-related and neurodegenerative diseases. MGBA serves as a bidirectional communication network coordinating brain and intestinal functions, in which the CNS interacts with gut microbiota primarily via microbially derived metabolites (Bravo et al., 2011; Yano et al., 2015). This conceptual evolution, from a bidirectional GBA to a tripartite MGBA, positions the gut microbiota not merely as a modulator but as a core regulator of brain function and, importantly, as a targetable node for therapeutic intervention. Given the established role of the MGBA in modulating brain function, it is plausible that high-altitude environments may exert neurocognitive effects through this axis.

High-altitude environments pose profound challenges to human physiology, with hypobaric hypoxia affecting multiple organ systems. Clinically, acute mountain sickness (AMS) exemplifies this connection. Characterized by headache, dizziness, nausea, vomiting, and anorexia, AMS simultaneously implicates both the central nervous system and the digestive tract (Gatterer et al., 2024). Beyond acute symptoms, prolonged high-altitude exposure leads to persistent neurocognitive impairments. Beyond AMS, emerging evidence suggests that MGBA disruption may underlie more persistent neurocognitive impairments under stress conditions. Microbial metabolites can cross the blood–brain barrier (BBB) and act directly on the brain (Haghikia et al., 2015; Guerrero Aznar et al., 2022). Specific genera such as *Bifidobacterium* and *Lactobacillus* have been shown to alleviate anxiety and depression while improving memory, learning, and cognition in various animal models (Sun et al., 2018; Varela-Trinidad et al., 2022). Conversely, gut dysbiosis, exacerbated by factors such as high-fat diet, can enhance systemic and central inflammatory responses, increasing susceptibility to Alzheimer’s disease pathology and cognitive decline via MGBA disruption (Bruce-Keller et al., 2015). These findings collectively suggest that modulation of the MGBA may represent a promising strategy for preventing brain dysfunction during high-altitude exposure. Direct evidence confirms that high-altitude environments alter intestinal microbiota balance, compromise intestinal structure and mucosal barrier integrity, and ultimately induce or exacerbate intestinal damage (Wang et al., 2022). A study finds that the relative abundance of *Blautia A* exhibited altitude-dependent changes, demonstrating that gut microbiota may contribute to inter-individual variability in responses to high-altitude exposure (Su et al., 2024). These observations support the plausibility of targeting the MGBA for neuroprotection under high-altitude conditions; however, systematic synthesis of the underlying mechanisms and therapeutic evidence is urgently needed.

**Figure 1:**
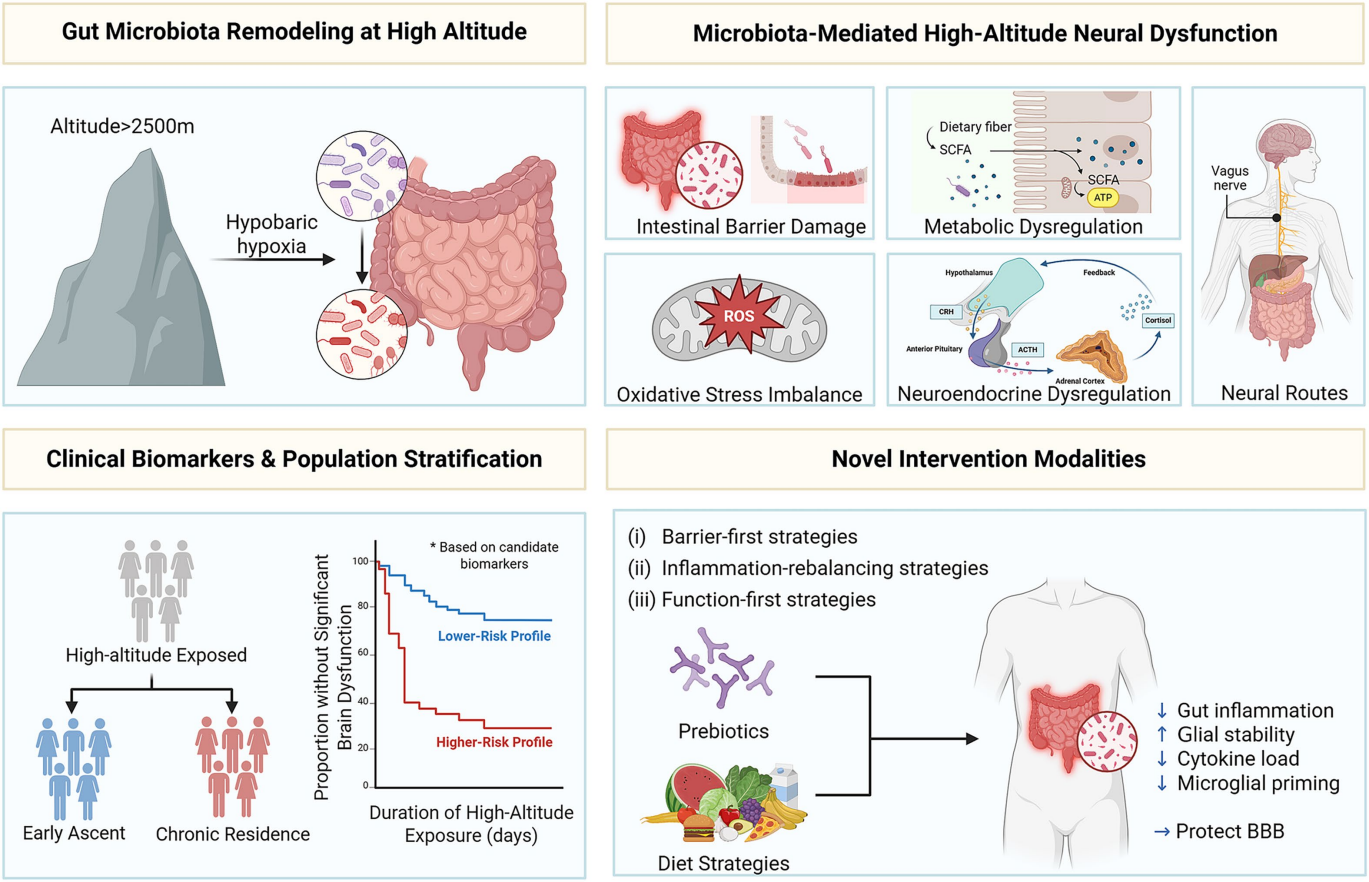
Microbiota-gut-brain axis dysfunction and neuroprotective strategies at high altitude. High-altitude hypoxia remodels gut microbiota, proposed to drive multiple key pathogenic mechanisms: intestinal barrier damage, metabolic dysregulation, vagal and enteric nervous system alterations, oxidative stress, and neuroendocrine imbalance, which maylead to cognitive impairment. Clinical biomarkers enable risk stratification. Targeted interventions (barrier-first, inflammation-rebalancing, function-first) protect blood–brain barrier integrity and neuroimmune function. FMT, fecal microbiota transplantation; BBB, blood–brain barrier. The lower-left panel conceptually illustrates how candidate biomarkers might stratify individuals by estimated cognitive stability over days at altitude.

In view of the close link between high-altitude exposure, gut microbiota disturbance, and subsequent neural dysfunction via the MGBA, further systematic elaboration and integration of related evidence are urgently required. Accordingly, the present review synthesizes current evidence on the potential roles of the MGBA in the pathophysiology of high-altitude-related brain impairment. Specifically, this review outlines the reshaping patterns of the gut microbiota in high-altitude environments, discusses the putative mechanisms by which gut dysbiosis may be associated with neural dysfunction, summarizes microbiota-targeted neuroprotective strategies, and provides perspectives on future research directions and clinical translation potential (see Figure 1).

## 2 Review methodology

This manuscript is a narrative review designed to synthesize and integrate current knowledge on MGBA in the context of high-altitude hypobaric hypoxia and brain health. Given the broad and emerging nature of this field, a narrative approach was chosen to provide a conceptual framework that encompasses taxonomic remodeling, mechanistic pathways, and therapeutic strategies, rather than to perform a quantitative meta-analysis.

### 2.1 Search strategy

We searched PubMed, Web of Science, and Cochrane for articles published up to March 2026. The search terms included combinations of the following keywords: *“high altitude,” “hypobaric hypoxia,” “gut microbiota,” “microbiome,” “microbiota-gut-brain axis,” “cognitive impairment,” “neuroinflammation,” “intestinal barrier,” “probiotics,” “prebiotics,”* and *“fecal microbiota transplantation.”* Reference lists of retrieved articles were also manually screened to identify additional relevant studies.

### 2.2 Inclusion and exclusion criteria

We included peer-reviewed original research articles (both animal and human studies), systematic reviews, and meta-analyses that directly addressed the interplay between high-altitude/hypoxia exposure, gut microbiota alterations, and neurological outcomes. Case reports, conference abstracts, and non-English articles were excluded. Because the literature on this topic remains relatively limited and heterogeneous, we did not impose strict restrictions on sample size or study duration, but we explicitly note the limitations of small or observational studies throughout the text.

### 2.3 Level of evidence

Throughout the review, we categorize evidence by study type (animal vs. human) and design (observational vs. interventional; summarized in Table 1). Current evidence is predominantly low-to-moderate: human data rely heavily on small, single-center observational studies, while mechanistic and intervention findings are mostly from preclinical rodent models with limited translational relevance.

**Table 1:** Summary of included studies: design, species, sample size, and environment.

Reference	Study design	Subjects / species	Sample size (n)	Environment
Part A: human clinical evidence				
Su et al. (2024)	Observational	Human (Han)	45	Real High-Altitude
Jia et al. (2020)	Observational	Human (Han & Tibetan)	393	Real High-Altitude
Han et al. (2024)	Observational	Human (Han & Tibetan)	610	Real High-Altitude
Zhang et al. (2023)	Observational	Human (Han & Tibetan)	147	Real High-Altitude
Huang et al. (2025)	Observational	Human (Han)	47	Real High-Altitude
Zhou Y. et al. (2025)	Observational	Human (Han)	230	Real High-Altitude
Li and Zhao (2015)	Observational	Human (Han & Tibetan)	35	Real High-Altitude
Lan et al. (2017)	Observational	Human (Tibetan)	208	Real High-Altitude
Li et al. (2016)	Observational	Human (Han & Tibetan)	68	Real High-Altitude
Simonson et al. (2010)	Observational	Human (Tibetan)	/	Real High-Altitude
Lorenzo et al. (2014)	Observational	Human (Han & Tibetan)	212	Real High-Altitude
Obregon-Tito et al. (2015)	Observational	Human (Others)	79	Sea Level / Normoxia
Li et al. (2018a)	Observational	Human (Tibetan)	24	Real High-Altitude
De Filippo et al. (2010)	Observational	Human (Others)	29	Sea Level / Normoxia
Yuan et al. (2023)	Observational	Human (Han & Tibetan)	207	Real High-Altitude
Kan et al. (2026)	Observational	Human (Han & Tibetan)	70	Real High-Altitude
Shen et al. (2020)	Observational	Human (Han)	111	Real High-Altitude
Lili et al. (2024)	Observational	Human (Ethnicity not Specified)	1 (Case Study)	Real High-Altitude
Fan et al. (2023)	Observational	Human (Han)	98	Real High-Altitude
Zhou W. et al. (2025)	Observational	Human (Han)	109	Real High-Altitude
Kleessen et al. (2005)	Observational	Human (Others)	7	Real High-Altitude
Dinmore et al. (1985)	Observational	Human (Others)	14	Real High-Altitude
Xie et al. (2023)	Observational	Human (Han & Tibetan)	80	Real High-Altitude
Strandwitz et al. (2019)	Observational	Human (Others)	23	Sea Level / Normoxia
Ma et al. (2025)	Observational	Human (Ethnicity not Specified)	406	Real High-Altitude
Karpęcka-Gałka et al. (2025)	Observational	Human (Others)	17	Real High-Altitude
Yu et al. (2025)	Interventional (RCT)	Human (Others)	17	Real High-Altitude
Part B: preclinical evidence				
Zhou Y. et al. (2025)	Interventional	Animal (Rat)	/	Simulated Hypobaric Chamber
Huang et al. (2026)	Observational	Animal (Yak)	/	Real High-Altitude
Li et al. (2018b)	Observational	Animal (Pika)	/	Real High-Altitude
Chi et al. (2019)	Observational	Animal (Bharals)	/	Real High-Altitude
Gao et al. (2020)	Observational	Animal (Tibetan Wild Ass)	/	Real High-Altitude
Zhang et al. (2018)	Interventional	Animal (Mice)		Sea Level / Normoxia
Wang et al. (2021)	Interventional	Animal (Mice)	/	Sea Level / Normoxia
Sakamuri (2023)	Interventional	Animal (Mice)	/	Sea Level / Normoxia
Zhu D. et al. (2022)	Interventional	Animal (Rats)	/	Real High-Altitude or in a Plain as Controls.
Qiu et al. (2026)	Interventional	Human-derived flora & cell lines	/	In vitro hypoxic model
Zhang et al. (2023)	Interventional	Animal (Mice)	/	Simulated Hypobaric Chamber
Gan et al. (2025)	Interventional	Animal (Mice)	/	Simulated Hypobaric Chamber
Colombo et al. (2021)	Interventional	Animal (Mice)	/	Sea Level / Normoxia
Peng et al. (2007)	Interventional	Caco-2 cell line	/	Sea Level / Normoxia
Zhou W. et al. (2025)	Interventional	Animal (Mice)	/	Simulated Hypobaric Chamber
Zeng et al. (2025)	Interventional	Animal (Mice)	/	Simulated Hypobaric Chamber
Qiao et al. (2025)	Interventional	Animal (Mice)	/	Simulated Hypobaric Chamber
Shang et al. (2025)	Interventional	Animal (Mice)	/	Simulated Hypobaric Chamber
Zhao et al. (2021)	Interventional	Animal (Mice)	/	Sea Level / Normoxia
Wei et al. (2025)	Interventional	Animal (Rats)	/	Sea Level / Normoxia
Chen A. et al. (2025)	Interventional	Animal (Rats)	/	Sea Level / Normoxia
Shi et al. (2026)	Interventional	Animal (Mice)	/	Simulated Hypobaric Chamber
Obrezchikova et al. (2000)	Interventional	Animal (Rats)	/	Simulated Hypobaric Chamber
Sharma et al. (2025)	Interventional	Animal (Rats)	/	Simulated Hypobaric Chamber
Xie et al. (2023)	Interventional	Animal (mice) & Cell lines	/	Simulated Hypobaric Chamber
Zhao et al. (2025)	Interventional	Animal (Mice)	/	Simulated Hypobaric Chamber
Freitas et al. (2014)	Interventional	Animal (Mice)	/	Sea Level / Normoxia
Strandwitz et al. (2019)	Interventional	Bacterial strains	/	Sea Level / Normoxia
Cuesta et al. (2022)	Interventional	Animal (mice) & Bacterial strains	/	Sea Level / Normoxia
Liu et al. (2022)	Interventional	Animal (Pigs)	/	Sea Level / Normoxia
Omaliko et al. (2024)	Interventional	Animal (Chickens)	/	Sea Level / Normoxia
Wang et al. (2023)	Observational	Animal (Rats)	/	Simulated Hypobaric Chamber
Bai et al. (2022)	Observational	Animal (Rats)	/	Simulated Hypobaric Chamber
Li et al. (2025)	Interventional	Animal (Mice)	/	Simulated Hypobaric Chamber
Braniste et al. (2014)	Interventional	Animal (Mice)	/	Sea Level / Normoxia
Erny et al. (2015)	Interventional	Animal (Mice)	/	Sea Level / Normoxia
Qiu et al. (2026)	Interventional	In vitro intestinal flora & cells	/	Simulated Hypobaric Chamber
Shi et al. (2026)	Interventional	Animal (Mice)	/	Simulated Hypobaric Chamber
Bakshi and Mishra (2025)	Interventional	Animal (Rats)	/	Simulated Hypobaric Chamber

Only original research studies are listed; reviews and commentaries are excluded. Sample sizes are summarized for human cohorts only. ‘Others’ indicates non-Chinese populations. ‘Ethnicity not Specified’ refers to Chinese cohorts where specific ethnic data (e.g., Han vs. Tibetan) were not explicitly detailed in the original text.

## 3 Remodeling of gut microbiota under high-altitude exposure

High-altitude hypoxia triggers a time-dependent restructuring of the gut microbial ecosystem. This section provides a multi-dimensional analysis of these dynamics, synthesizing evidence on alterations in alpha- and beta-diversity, phylum- and genus-level taxonomic shifts, and functional metabolic remodeling of the microbiota. The trajectory of microbial changes is closely tied to exposure duration, with early stages characterized by rapid taxonomic oscillations and a marked decline in diversity, followed by gradual stabilization and functional adaptation upon prolonged residence. A key feature of this adaptive process is the progressive convergence of the gut microbiota in Han migrants toward the microbial composition of native Tibetans, suggesting the establishment of a high-altitude-adapted microbial configuration. Notably, this altitude-induced microbial imprint exhibits remarkable persistence, with characteristic features remaining evident even months after return to lowland environments. Beyond the direct effects of hypoxia, the trajectory of microbial remodeling is modulated by complex interactions among host genetics, dietary patterns, and individual factors such as age and baseline microbiota composition. Together, these findings establish the gut microbiota as a highly dynamic and responsive system that plays an integral role in host acclimatization to high-altitude environments.

### 3.1 Trajectories of gut microbiota remodeling

#### 3.1.1 Alterations in alpha- and beta-diversity

High-altitude exposure induces rapid and dynamic changes in the diversity of the gut microbial ecosystem, with the trajectory of these changes closely tied to the duration of exposure. Upon acute ascent, the response can be heterogeneous. One longitudinal study reported no significant change in alpha diversity within the first 2 days, followed by a marked decrease in both Shannon and richness indices by day 24 of exposure (Su et al., 2024). Similarly, another study observed that alpha diversity in Han migrants was significantly lower after 6 days on the plateau compared to plain dwellers (Jia et al., 2020). This reduction becomes more pronounced with prolonged residence, as alpha diversity was significantly decreased after 6 months at high altitude compared to those who had stayed for only 1 week (Han et al., 2024). During extended exposure, diversity levels can exhibit partial recovery; for instance, the richness index may rebound while the Shannon index remains lower even after 66 days (Su et al., 2024), and diversity can remain relatively low after more than 3 months of residence (Jia et al., 2020). Interestingly, in a one-year follow-up study, the Shannon index of Han migrants was higher at 3 months than at 6 or 12 months, eventually returning to levels close to those of plain controls by the end of the year (Zhang et al., 2023). This pattern of change is not exclusive to migrants, as native Tibetans also showed a gradual decrease in the Chao1 index upon changing their living environment (Zhang et al., 2023).

Beta diversity analyses consistently demonstrate a significant restructuring of the microbial community. The composition of high-altitude migrants diverges significantly from that of lowland controls (Huang et al., 2025; Zhou Y. et al., 2025). Over time, the gut microbiota of Han migrants progressively converges toward that of native Tibetans, with the community distance being smallest after 6 months of cohabitation (Han et al., 2024). Intriguingly, intra-group variability decreases in the early stages of exposure, suggesting a convergence of community structures among individuals, which later recovers (Su et al., 2024). The impact of the high-altitude environment is persistent; the microbiota of individuals who returned to the plains for 3 months still clustered closer to those of current plateau residents than to their baseline lowland counterparts (Jia et al., 2020). Notably, most human findings derive from small observational cohorts, limiting causal inference and generalizability.

#### 3.1.2 Phylum-level instability and stabilization

At the phylum level, high-altitude exposure triggers significant compositional shifts. A higher abundance of Bacteroidetes and a lower abundance of Firmicutes have been observed in native Tibetans compared to plain-dwelling Han populations (Han et al., 2024; Li and Zhao, 2015). Consistently, the Firmicutes/Bacteroidota (F/B) ratio is significantly higher in lowland Han than in Tibetans (Lan et al., 2017).

Longitudinal studies reveal a dynamic process of instability followed by stabilization. In Han migrants, several phyla, including Proteobacteria, show an initial increase in abundance within the first 3 months, which then decreases over the subsequent months (Zhang et al., 2023). Conversely, Firmicutes A emerges as the dominant phylum during exposure, demonstrating a significant increase by the end of the exposure period, a finding validated in independent long-term cohorts (Su et al., 2024). Other notable shifts include a consistent increase in Fusobacteria over a 12-month period (Zhang et al., 2023) and alterations in non-bacterial phyla, such as an increase in the archaeal phylum Crenarchaeota and a decrease in Thaumarchaeota after 12 months of moderate altitude exposure (Huang et al., 2025).

#### 3.1.3 Genus-level dynamics

The genus-level dynamics under high-altitude environment are characterized by the depletion of some taxa and the enrichment of others, ultimately driving a convergence toward high-altitude-adapted microbial composition. Genera such as *Bifidobacterium*, *Bacteroides*, *Agathobacter*, and *Collinsella* are often found in lower abundance in plateau populations compared to lowland controls (Jia et al., 2020; Zhang et al., 2023; Li et al., 2016). In contrast, genera like *Prevotella*, *Succinivibrio*, *Roseburia*, and *Phascolarctobacterium* are enriched in high-altitude populations, with *Prevotella* and *Succinivibrio* being particularly prominent in native Tibetans (Zhang et al., 2023; Huang et al., 2025). The enrichment of *Roseburia* in plateau migrants persists even after returning to the plains, whereas the levels of *Butyricimonas* appear more sensitive and revert upon descent (Jia et al., 2020).

The most striking example of a genus-level adaptive response is *Blautia A*. Its relative abundance increases dramatically during high-altitude exposure, rising from a low baseline to become the most dominant genus, and this elevation is sustained in long-term high-altitude cohorts (Su et al., 2024). This genus was identified as a key orchestrator of microbial community changes, with nearly half of all indicator species in the early exposure phase belonging to *Blautia A* (Su et al., 2024). Upon return to low altitude, its abundance declines rapidly (Su et al., 2024). Other taxa show distinct patterns related to health status. In individuals with high-altitude heart health abnormalities, a panel of species including *Streptococcus rubneri* and several *Veillonella* species are significantly depleted, while others like *Campylobacter jejuni* are enriched (Zhou Y. et al., 2025).

A substantial number of species show altitude-dependent reversibility. For instance, 57 species, including butyrate producers like *Faecalibacterium prausnitzii*, are depleted during high-altitude acclimatization but rebound upon return to low altitude (Han et al., 2024). Conversely, 51 species like *Prevotella copri* are enriched during acclimatization with purported health benefits (Han et al., 2024).

#### 3.1.4 Functional and metabolic remodeling of the gut microbiota

The taxonomic shifts are underpinned by profound functional and metabolic remodeling of the gut microbiome. A key adaptive feature is the increase in functional redundancy (FRα) under prolonged hypoxia, which helps maintain community stability despite a decrease in functional diversity, and this elevation in FRα persists even after returning to low altitudes (Su et al., 2024; Li et al., 2021).

Metagenomic analyses have identified specific pathways that are consistently altered. Pathways related to “cofactor and vitamin metabolism” and “energy metabolism,” particularly cobalamin biosynthesis and methanogenesis, are significantly enriched during exposure, with *Blautia A* species playing a central role in these processes (Su et al., 2024). Pan-genomic analysis further confirms that *Blautia A* is equipped with the enzymatic machinery for butyrate production, primarily by regulating pyruvate metabolism (Su et al., 2024; Liu X. et al., 2021). In contrast, pathways related to the citrate cycle, ubiquinone biosynthesis, and heme biosynthesis are downregulated in both acclimatized Han migrants and Tibetans, suggesting an inhibition of aerobic respiration in the gut microbial community under hypoxia (Han et al., 2024). This is accompanied by reduced microbial capacity for the biosynthesis of several amino acids and the downregulation of GABA production (Han et al., 2024).

Metabolic pathway alterations are also linked to host health outcomes. In individuals with heart health abnormalities, microbial functions related to oxidative phosphorylation and fatty acid metabolism are significantly enriched (Zhou Y. et al., 2025; Ritterhoff and Tian, 2017; Murray, 2016). Integrated analysis revealed that the depletion of protective species like *S. rubneri* and *V. rogosae* in these individuals is associated with negative effects on key enzymes of the TCA cycle and oxidative phosphorylation, potentially shifting host energy metabolism toward glycolysis (Zhou Y. et al., 2025). Furthermore, purine metabolism is remodeled at altitude, with enhanced inosine monophosphate (IMP) biosynthesis and inhibited purine degradation, changes that correlate with specific taxa and are linked to host plasma uric acid levels (Han et al., 2024). Finally, a wide array of specific metabolic pathways are altered after 12 months of moderate altitude exposure, with upregulation of pathways like xyloglucan degradation and downregulation of pathways like tetrahydrofolate biosynthesis, driven by distinct bacterial groups (Huang et al., 2025).

### 3.2 Modulating factors: host-environment-microbiota interactions

#### 3.2.1 Host genetics

Studies have revealed that natural selection in Tibetans has acted on multiple loci involved in hypoxia response, most notably endothelial PAS domain protein 1 (EPAS1) and egl nine homolog 1 (EGLN1) in the hypoxia-inducible factor (HIF) pathway (Simonson et al., 2010; Lorenzo et al., 2014), which are also associated with distinct gut microbial configurations (Li and Zhao, 2015; Lan et al., 2017). This raises the possibility that host genetic adaptation to high-altitude hypoxia may shape the intestinal microbial ecosystem by modulating physiological traits such as erythropoiesis, vascular tone, and energy metabolism. In addition to hypoxia-responsive genes, positively selected genes involved in energy metabolism have been identified in Tibetan genomes (Liu K. et al., 2021). Given that gut microbiota contribute to host energy harvest via SCFA production, these genetic adaptations may functionally converge with microbial metabolic activities to optimize energy utilization under hypoxic conditions. Beyond compositional differences, the functional output of the MGBA is profoundly shaped by cell-type-specific metabolic dialogs at the epithelial interface—a process that may itself be influenced by host genetic variation. Recent findings in high-altitude-adapted yaks illustrate that specific microbial strains can influence the accumulation of key metabolites such as succinate and lactic acid by modulating the metabolic activities of distinct epithelial cell types. This process involves intercellular communication mediated by key receptors like SLC27A5 and PPARA, which subsequently reprogram core energy pathways including glycolysis and the TCA cycle (Huang et al., 2026). Such a mechanism suggests that the adaptability of the gastrointestinal tract to extreme environments may depend not only on the composition of the microbiota but also on the host’s ability to engage in fine-tuned, cell-type-specific metabolic crosstalk with resident microorganisms.

#### 3.2.2 Diet-microbiota crosstalk

Urbanization on the plateau dramatically alters dietary patterns and gut microbiota. Traditional Tibetan herders, consuming high-fiber foods (Zanba, dairy), exhibit Prevotella-dominated microbial composition, while urbanized Tibetans (consuming more noodles and rice) show increased Bacteroides, Faecalibacterium, and Blautia, resembling lowland urban populations (Obregon-Tito et al., 2015; Li et al., 2018a; Li et al., 2018b). This shift reflects the loss of fiber-degrading capacity and may have implications for metabolic health and adaptation efficiency (De Filippo et al., 2010). Captive breeding of wildlife also illustrates the impact of diet on microbiota: captive animals fed artificial fodder show reduced microbial diversity, increased pathogens, and enrichment of antibiotic resistance genes, compromising their adaptability and health (Chi et al., 2019; Gao et al., 2020). These findings underscore the plasticity of gut microbiota in response to dietary changes (Liu K. et al., 2021). The profound impact of dietary patterns on gut microbiota composition underscores the potential of nutritional interventions as a scalable strategy to support high-altitude adaptation and metabolic health.

#### 3.2.3 Individual variability

Individual factors such as age, sex and migration duration, and baseline microbiota composition modulate the response to high-altitude exposure (Jia et al., 2020; Yuan et al., 2023; Jia et al., 2025; Kan et al., 2026). A recent longitudinal study of healthy adults ascending to 4,500 m further revealed that the trajectory of microbial and metabolic remodeling during high-altitude acclimatization differs between young and middle-aged individuals, underscoring the role of age as a key modulator of host-microbiota responses to hypoxic stress (Kan et al., 2026). Beyond age, sex is emerging as a critical modulator of physiological responses to high-altitude hypoxia (Vento et al., 2022; Burtscher et al., 2024). A growing body of evidence suggests that women may exhibit higher susceptibility to acute mountain sickness (AMS) compared to men under comparable ascent conditions. In a prospective study of 99 healthy lowlanders ascending from 500 m to 4,100 m, the incidence of AMS was 75% in women vs. 34% in men, with female sex emerging as the strongest independent risk factor (OR = 6.32, *p* < 0.001; Shen et al., 2020). However, the same study revealed that a larger reduction in SpO₂ after exercise at low altitude—a validated AMS predictor in men—was not significantly associated with AMS incidence in women, indicating that predictive physiological markers may be sex-dependent (Shen et al., 2020). These findings underscore that sex differences are not merely a matter of degree but may involve qualitatively distinct pathophysiological pathways, which has profound implications for the development of personalized risk stratification and prevention strategies in high-altitude medicine. Although direct evidence remains scarce, studies in other hypoxia or stress contexts indicate that sex hormones can influence gut microbial composition, intestinal barrier function, and neuroinflammatory outcomes (Zhang et al., 2018; Wang et al., 2021; Sakamuri, 2023; Ley and Saha, 2025; Stahlke and Theiss, 2025). For instance, estrogen has been shown to regulate tight junction proteins and exert anti-inflammatory effects on the gut–brain axis, potentially conferring sex-specific resilience or vulnerability to hypoxic insult (Wang et al., 2021; Zhu D. et al., 2022; Hoyer-Kimura et al., 2023). Future high-altitude studies should therefore stratify analyses by sex, report sex-disaggregated data, and, where sample size permits, explicitly test for sex-by-environment interactions in MGBA-mediated outcomes. To date, most preclinical high-altitude studies have predominantly used male rodents, leaving female responses largely unexplored. This gap limits the generalizability of current mechanistic models. Longitudinal studies of healthy Han Chinese adults revealed that high-altitude exposure rapidly (within 4 days) and persistently (more than 3 months) reshapes both gut microbiota composition and blood clinical parameters (Jia et al., 2020). With prolonged residence at high altitude, the gut microbiota of Han migrants progressively converges toward the characteristic features of the native Tibetan population. Notably, this altitude-induced microbial imprint persists even after returning to low altitude for 3 months, indicating that the hypoxic environment leaves a lasting signature on the human microbiome (Jia et al., 2020). These findings underscore the profound and enduring impact of high-altitude hypoxia on gut microbial ecology and highlight the importance of considering exposure duration and individual baseline variation in future studies, which should stratify by these variables to develop personalized interventions (Lili et al., 2024). Understanding how these individual factors modulate the trajectory of microbial remodeling is critical for identifying populations at heightened risk for MGBA dysfunction and for designing personalized preventive strategies.

### 3.3 From dysbiosis to pathogenic cascade

**Figure 2:**
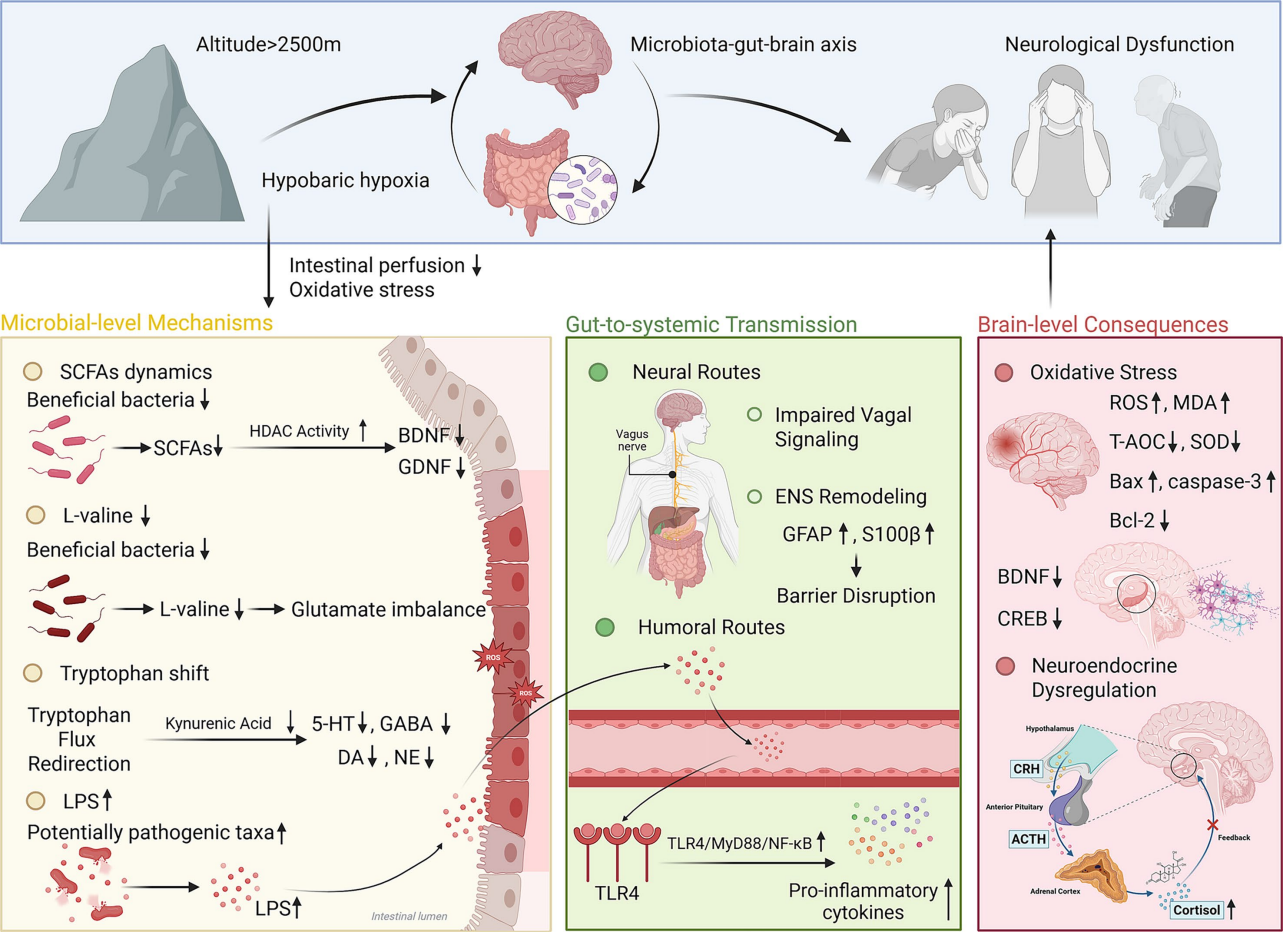
Proposed pathophysiological mechanisms of microbiota-gut-brain axis dysfunction under high-altitude hypoxia. The flow diagram illustrates the putative cascades from environmental hypoxia to cognitive decline across three interactive levels, based largely on associative preclinical evidence. At the microbial level, hypoxia-induced dysbiosis leads to SCFA reduction (hypothesized to upregulate HDAC activity), L-valine depletion, a putative tryptophan metabolic shift, and mucosal LPS translocation. Through humoral (circulating endotoxins and metabolites) and neural (impaired vagal signaling) routes, these peripheral signals communicate with the CNS. Consequently, at the brain level, neuroinflammation, BBB disruption, oxidative stress, and synaptic dysfunction are triggered. This is further compounded by neuroendocrine dysregulation resulting from the proposed disruption of the HPA axis feedback loop, ultimately driving central cognitive deficits.

These findings collectively demonstrate that high-altitude hypoxia induces profound structural and functional remodeling of the gut microbiota, establishing a dysbiotic state that serves as both a consequence of environmental stress and an upstream trigger of neural dysfunction. The observed microbial alterations, ranging from taxonomic depletion to metabolic pathway disruption, create a permissive environment for a cascade of pathogenic events along the MGBA. In the following section, we systematically dissect how these dysbiosis-induced perturbations activate five core mechanisms that converge to compromise neurological integrity (see Figure 2).

## 4 Putative mechanisms linking gut microbiota dysbiosis to high-altitude neural dysfunction via the MGBA

High-altitude hypobaric hypoxia is a typical extreme environmental stressor that disrupts the homeostasis of the host gut microbiota. It has been hypothesized that subsequent gut microbial dysbiosis may contribute to neural dysfunction through the GBA. However, most available evidence is correlational, and it remains possible that microbiota changes represent adaptive or secondary responses to hypoxia rather than primary pathogenic drivers. This bidirectional regulatory axis connects the intestinal tract and the central nervous system (CNS) through neural, immune, endocrine, and metabolic pathways. Under high-altitude hypoxia, alterations in gut microbial composition and function have been associated with intestinal barrier damage and may trigger a cascade of pathological reactions such as systemic inflammation, metabolic disorder, and oxidative stress. These reactions could, in theory, be transmitted to the brain through the MGBA, potentially leading to neuroinflammation, synaptic plasticity damage, and neurotransmitter dysregulation, which have been linked to cognitive impairment, working memory decline, and emotional disorders. The following sections describe five interconnected mechanisms that have been proposed to mediate this relationship, based largely on preclinical models and associative human studies. Of note, most mechanistic data come from rodent hypobaric hypoxia models, which do not fully recapitulate real high-altitude conditions (e.g., cold, UV). Rodents also differ from humans in gut microbiome composition and immune responses, limiting direct translational relevance.

### 4.1 Microbial-level mechanisms: functional and metabolic dysregulation

Gut microbiota is an important “metabolic organ” of the host, and its dysbiosis under high-altitude hypoxia conditions has been linked to alterations in key metabolite synthesis and metabolic pathways, which may affect the normal physiological function of the CNS through the MGBA, and could represent a mechanism contributing to neural dysfunction. Short-chain fatty acids (SCFAs), L-valine, and tryptophan metabolites are the core microbial metabolites involved in this regulatory process, and their abnormal synthesis or metabolic flow has been proposed to contribute to neural function dysfunction under hypoxic stress.

#### 4.1.1 SCFA dynamics and neurotrophic signaling under high-altitude hypoxia

SCFAs are key metabolites of beneficial gut bacteria, and their availability under high-altitude hypoxia may be influenced by shifts in SCFA-producing taxa: while several genera such as *Bifidobacterium*, *Agathobacter*, and *Collinsella* are depleted in Han migrants during high-altitude exposure, the SCFA-producing families *Ruminococcaceae* and *Lachnospiraceae*, along with microbial *BCoAT* gene abundance, are enriched in high-altitude-adapted populations (Zhao et al., 2024). This dual pattern suggests that an expanded butyrate-producing capacity may represent part of the adaptive response to chronic hypoxia, while transient SCFA insufficiency during acute exposure or in poorly acclimatized individuals could still permit neural dysfunction to emerge.

Among SCFAs, butyrate has been most extensively studied in the context of hypoxia and shows two complementary actions. First, it can dampen hypoxia-induced HIF-1α accumulation. In a high-altitude rat model and in CoCl₂-treated NCM460 colonic epithelial cells, butyrate reduced HIF-1α protein levels, accompanied by suppressed LDHA enzymatic activity and decreased intracellular lactate (Zhao et al., 2024). The authors proposed that lactate reduction may relieve PHD2-mediated stabilization of HIF-1α, though direct empirical validation remains limited (Zhao et al., 2024). A convergent reduction in HIF-1α was observed in CoCl₂-treated PC12 cells exposed to SCFA-enriched fermentation metabolites of plateau-derived gut microbiota, in that case associated with upregulation of PHD, FIH, and VHL (Qiu et al., 2026), although the two studies share no overlapping mechanistic readouts and the upstream pathway linking SCFAs to HIF-1α regulation in altitude contexts remains to be established. Second, it promotes neuronal survival through histone deacetylase (HDAC) inhibition, leading to histone hyperacetylation and increased expression of neuroprotective genes like brain-derived neurotrophic factor (BDNF) and glial cell-derived neurotrophic factor (GDNF; Rahmani et al., 2025). Although direct evidence for this HDAC–BDNF pathway in high-altitude models remains limited, it provides a plausible molecular link between SCFA availability and synaptic resilience under hypoxia.

Experimental evidence further suggests potential links between hypoxia-associated microbial alterations and impaired neurotrophic signaling. In mice exposed to simulated high-altitude conditions, exposure reduced the abundance of Lactobacillaceae, with hippocampal BDNF downregulation observed when this depletion became severe (Zhang et al., 2023). Because BDNF is an important regulator of synaptic plasticity and neuronal activity, these findings are consistent with the hypothesis that SCFA-producing taxa support hippocampal neurotrophic tone, and that their loss under hypoxia contributes to synaptic dysfunction.

However, the relationship between BDNF and neural function under chronic hypoxia appears complex: in long-term Han migrants at high altitude (mean exposure ~11 years), higher serum BDNF levels were associated with poorer executive control, a pattern that has been interpreted as a possible compensatory or self-rehabilitative response to hypoxia-induced neural stress (Fan et al., 2023; Gan et al., 2025). Such a pattern argues against interpreting BDNF as a unidirectional biomarker of brain health at altitude: in the acute phase, microbiota-supported BDNF appears protective, whereas chronically elevated BDNF may instead signal ongoing neural compromise. Disentangling protective from compensatory BDNF dynamics—and clarifying how SCFA-producing taxa modulate this trajectory across exposure phases—represents a key open question for MGBA-based neuroprotection strategies. Beyond the BDNF dynamics discussed above, SCFA action under hypoxia warrants similar caution, as three considerations argue against a uniformly protective view. First, butyrate has been reported to reduce HIF-1α protein levels under hypoxia (Zhao et al., 2024), this is not unambiguously beneficial: HIF-1α drives core cellular hypoxic adaptations including glycolytic reprogramming, suppression of mitochondrial respiration, and angiogenesis (Semenza, 2000). Yet naturally selected Tibetan genetic variants in *PHD2* and *HIF2A* appear to favor reduced rather than enhanced HIF activity (Bigham and Lee, 2014), indicating that the optimal HIF tone under chronic hypoxia is itself uncertain and likely depends on exposure timescale and host genetic background—so the net effect of butyrate-induced HIF attenuation is not predictable in advance. Second, in non-altitude neurodegenerative models, microbiota-id SCFAs activate microglia and promote amyloid-*β* deposition (Colombo et al., 2021), indicating that their effect on glial cells is not invariably anti-inflammatory. Third, butyrate’s action on the intestinal epithelium is concentration-dependent and reverses direction across the physiological range, strengthening tight junctions at low millimolar levels but compromising barrier integrity at higher concentrations (Peng et al., 2007). SCFAs should therefore be viewed as a conditionally protective signal—likely beneficial when restored from deficiency, but with uncertain effects when supplemented on top of an already-adapting host—and altitude studies should jointly report SCFA flux, HIF-1α readouts, glial activation, and barrier integrity rather than inferring net benefit from SCFA levels alone.

#### 4.1.2 L-valine malabsorption and hippocampal glutamate-BDNF deficits

In a cohort of young Han migrants at high altitude, *Clostridium* abundance was generally elevated relative to lowland controls but was significantly lower in migrants with cognitive impairment than in those with preserved cognition, suggesting that *Clostridium* enrichment may be part of a successful adaptive response and that its relative deficiency tracks adaptation failure. This taxonomic shift was accompanied by reduced serum and elevated fecal L-valine concentrations, consistent with impaired intestinal absorption of this essential amino acid. Mechanistic studies in hypobaric hypoxia-exposed mice indicate that hypoxia compromises the intestinal barrier, providing a plausible route by which L-valine absorption is disrupted. *Clostridium* sp. supplementation in these mice restored barrier integrity, increased fecal SCFAs, enhanced L-valine absorption, and raised hippocampal levels of L-valine, BDNF, and glutamate, thereby mitigating neuroinflammation, synaptic ultrastructural damage, and cognitive deficits. Of note, the direct evidence linking barrier dysfunction to L-valine malabsorption derives from the mouse model rather than the human cohort, so the integrated pathway should be regarded as mechanistically plausible but not yet established in humans (Zhou W. et al., 2025).

#### 4.1.3 Tryptophan metabolism skew and neurotransmitter precursors

The microbial catabolism of tryptophan represents a key functional hub along the MGBA under high-altitude stress. In C57BL/6 J mice exposed to simulated hypobaric hypoxia, chronic oxygen deprivation significantly impaired spatial learning and memory retention. Integrated multi-omics analysis revealed that this cognitive decline strongly correlated with a shift in tryptophan metabolism. Specifically, escape latency extension in the Morris water maze was positively correlated with higher fecal tryptamine but negatively correlated with xanthurenic acid (a kynurenine-pathway intermediate). This pattern underscores a hypoxia-associated alteration in tryptophan metabolism. Specifically, the opposing correlations of tryptamine and xanthurenic acid imply a relative redirection of peripheral tryptophan flux Notably, microbial depletion via antibiotics further worsened memory retention deficits, yet it did not further reduce hippocampal BDNF, synaptophysin (SYP), or postsynaptic density protein 95 (PSD-95) expression (Zeng et al., 2025). Dietary context may further broaden and complicate this neuropathology. In mice exposed to high-fat diet under hypoxic conditions, metabolomic and KEGG analyses converged on tryptophan–kynurenine pathways, characterized by a significant reduction of kynurenic acid and 2-oxoadipic acid in the colon, as well as D-kynurenine in the hippocampus. This compartmentalized depletion was accompanied by lowered cerebral 5-HT, norepinephrine, dopamine, and GABA, alongside pronounced depressive/anxiety-like behaviors (Qiao et al., 2025). Because diet and hypoxia were applied together, their relative contributions cannot be completely disentangled in this cross-sectional design. However, the consistent convergence on kynurenine-pathway alterations—whether under extreme simulated hypobaric hypoxia alone or moderate hypoxia coupled with dietary stressors—reinforces this metabolic axis as a highly recurrent microbiota-linked target under high-altitude environmental stress.

### 4.2 Gut-to-systemic transmission

#### 4.2.1 Humoral routes: intestinal barrier damage and LPS/TLR4-mediated inflammation

High-altitude hypobaric hypoxia can reduce splanchnic perfusion, a phenomenon further exacerbated by physical exertion (McKenna et al., 2022). The resulting local tissue hypoxia and oxidative stress are proposed to compromise the intestinal barrier, potentially altering tight junction integrity and mucosal structures (Kleessen et al., 2005; McKenna et al., 2022). While these stressors are hypothesized to increase paracellular permeability under acute or severe exposure, human data remain inconsistent. For instance, a classic study by Dinmore et al. at 5,730 m demonstrated that while active mucosal absorption of monosaccharides was substantially reduced, lactulose/L-rhamnose permeability ratios remained statistically unchanged from sea-level baselines (Dinmore et al., 1985). This suggests that altitude-induced hypoxia may impair active nutrient transport prior to, or independently of, driving macroscopic paracellular barrier failure in humans. Concurrently, high-altitude stressors are associated with shifts in gut microbial ecology, although human and animal empirical findings vary (Kleessen et al., 2005; Shang et al., 2025). In human mountaineers, high-altitude exposure can suppress specific beneficial taxa, such as *Bifidobacterium*, and compromise systemic immune tolerance against endotoxins, as evidenced by significant declines in anti-lipopolysaccharide (anti-LPS) antibodies (Kleessen et al., 2005). Furthermore, preclinical evidence demonstrates that specific diets, such as a high-carbohydrate regimen under chronic hypoxia, can amplify this dysbiosis (Shang et al., 2025). This shift favors the proliferation of opportunistic pathogens and augments LPS release, forming a pathogenic cascade that further reinforces epithelial barrier vulnerability (Shang et al., 2025).

In rodent models of simulated hypobaric hypoxia, the disrupted intestinal mucosal integrity and compromised tight junctions facilitate the leakage of lumen-derived LPS into the peripheral circulation, driving the development of systemic endotoxemia (Zhou W. et al., 2025). As a pivotal pro-inflammatory signaling molecule, circulating LPS can bind to Toll-like receptor 4 (TLR4) expressed on the surface of immune cells, intestinal epithelial cells, and central nervous system cells such as microglia, thereby activating the downstream TLR4/MyD88/NF-κB signaling pathway (Zhao et al., 2021). Within the conceptual framework of high-altitude neurological impairment, this classic cascade is highly prioritized, as hypoxia-induced systemic challenges are hypothesized to leverage these generalized immune-inflammatory pathways to drive secondary neuroinflammatory responses and cognitive deterioration (Zhao et al., 2021; Wei et al., 2025; Chen A. et al., 2025). This pathway activation can induce a large number of pro-inflammatory cytokines (TNF-*α*, IL-6, IL-1β, IFN-*γ*) to be released in the intestinal tract, peripheral circulation, and even the CNS, thereby establishing a pathological feed-forward loop (Zhang et al., 2023; Shi et al., 2026). Specifically, the resultant local intestinal inflammation further compromises mucosal barrier integrity, facilitating sustained translocation of LPS and inflammatory factors into the bloodstream. Concurrently, elevated circulating LPS and systemic cytokine loads propagate these inflammatory signals to the CNS. This peripheral-to-central transmission is mirrored by a concurrent increase in hippocampal endotoxin and inflammatory levels, which strongly correlate with microglial activation and neuronal injury in mice (Zhou W. et al., 2025).

Valuable mechanistic insights into this pathway can be drawn from other hypoxic context frameworks; for example, in neonatal HIBD models, intestinal *Enterobacteriaceae* overgrowth is associated with increased LPS levels, which activate TLR4-mediated inflammatory signaling, correlating with hippocampal neuronal damage and cognitive impairment (Wei et al., 2025). While both environmental settings differ in clinical pathology, this classic signaling cascade in neonatal models parallels the observations in high-altitude hypobaric hypoxia contexts. This mechanistic overlap underscores the necessity of investigating the precise causal role of the LPS-TLR4 axis under high-altitude conditions, positioning it as a highly prospective therapeutic link between gut dysbiosis and neural dysfunction.

#### 4.2.2 Neural routes: vagal afferent signaling and the enteric nervous system

The vagus nerve and its main central projection site, the nucleus tractus solitarii (NTS), constitute essential pathways for bidirectional gut-brain communication (Kinkead et al., 2019). Emerging evidence from hypoxic models indicates that environmental oxygen deprivation affects multiple neural components along this axis.

At the central vagal reflex level, periodic high-altitude hypoxia significantly attenuates baroreflex vagal bradycardia in rats, whereas peripheral responses to direct vagal electrical stimulation or muscarinic agonists remain unaltered (Obrezchikova et al., 2000). This implies that hypoxia selectively impairs the central processing elements of the reflex arc rather than peripheral components (Obrezchikova et al., 2000). Whether such central desensitization also affects the processing of gastrointestinal vagal afferents—and thereby potentially influences microbiota-gut-brain signaling—remains to be determined.

Conversely, ascending neural pathways show potential for therapeutic modulation against hypoxic injuries. In normobaric hypoxia models, vagus nerve stimulation (VNS) delivered during hypoxia exposure ameliorates deficits in passive avoidance learning (Sharma et al., 2025). This cognitive rescue correlates with partial or complete restoration of hypoxia-induced downregulations of hippocampal NGF and BDNF at both mRNA and protein levels (Sharma et al., 2025).

Parallel to central alterations, the peripheral enteric nervous system (ENS) undergoes functional remodeling. High-altitude exposure elevates serum biomarkers of enteric glial cells (EGCs) in humans and upregulates glial fibrillary acidic protein and S100β expression in the mouse small intestine (Xie et al., 2023). These glial markers correlate negatively with intestinal tight junction proteins, and hypoxic EGC-conditioned medium or exogenous S100β directly reduces tight junction expression in epithelial cells *in vitro* (Xie et al., 2023).

Collectively, these findings demonstrate that hypoxia modulates the gut-brain neural circuitry at central reflex sites, responds to afferent neuromodulation, and drives EGC-mediated barrier disruption. However, direct evidence establishing that high-altitude gut microbial dysbiosis leverages these vagal afferent or ENS pathways to cause neural dysfunction is still lacking, necessitating future prospective investigations using vagotomy and neural-tracing approaches.

### 4.3 Brain-level consequences

#### 4.3.1 Oxidative stress in the CNS

High-altitude hypobaric hypoxia and accompanying gut microbial dysbiosis jointly contribute to oxidative stress imbalance, serving as a potential amplification mechanism in neural dysfunction. At the gastrointestinal level, exposure to hypobaric hypoxia directly induces localized oxidative stress in intestinal epithelial cells, characterized by accelerated reactive oxygen species production, elevated systemic endotoxin levels, and increased bacterial translocation (Qi et al., 2025). This peripheral barrier failure may subvert systemic redox homeostasis, potentially cascading into central oxidative damage. In preclinical models, chronic high-altitude hypoxia exposure significantly increases malondialdehyde (MDA) content while impairing antioxidant defense systems, as evidenced by reduced total antioxidant capacity (T-AOC) and suppressed superoxide dismutase (SOD) activities in both serum and whole brain tissues of mice (Zhao et al., 2025).

Region-specific investigations further elucidate these hypoxic brain injuries. In the prefrontal cortex, high-altitude-induced gut microbiota disruption correlates with diminished T-AOC, heightened MDA accumulation, and altered activities of SOD and glutathione peroxidase (GSH-Px; Zhao et al., 2022). These localized oxidative shifts occur concurrently with upregulation of pro-apoptotic cascades, specifically featuring elevated expression of Bax and caspase-3 alongside downregulation of anti-apoptotic Bcl-2 (Zhao et al., 2022). Because an increased Bax-to-Bcl-2 ratio represents a well-established molecular marker for enhanced vulnerability to apoptotic activation (Freitas et al., 2014), interventions that directly modulate the disrupted intestinal flora under high-altitude stress have been shown to mitigate these prefrontal oxidative damages and neuronal apoptotic pathways (Zhao et al., 2022).

Distinct pathological alterations are also manifested within the hippocampal formation. Extreme high-altitude conditions directly induce oxidative damage in the hippocampus, thereby destabilizing synaptic plasticity (Gan et al., 2025). This neuroinjury is mediated by the disruption of cAMP-response element-binding protein (CREB) activation and brain-derived neurotrophic factor (BDNF) expression (Gan et al., 2025). Accumulating evidence indicates that gut microbial dysbiosis-induced systemic inflammation is closely associated with this elevated central oxidative stress, potentially creating a reciprocal pathogenic loop that exacerbates neuronal injury and functional deficits (Zhao et al., 2022; Bruce-Keller et al., 2015). Confirming the viability of targeting this peripheral-central axis, probiotic intervention with *Lactobacillus johnsonii* HL79 significantly fortifies antioxidant responses in mice subjected to hypobaric hypoxia (Zhao et al., 2025). This targeted microbial therapy effectively decreases MDA content and restores SOD and catalase activities in both serum and whole brain tissues, which is accompanied by alleviation of hippocampal synaptic dysfunction and corresponding cognitive impairment (Gan et al., 2025; Zhao et al., 2025). Taken together, these findings support the involvement of oxidative stress imbalance as a core mechanistic link in gut microbial dysbiosis-mediated high-altitude neural injury.

#### 4.3.2 Neuroendocrine dysregulation and HPA axis activation

The gut microbiota has been reported to influence the host’s neuroendocrine system and CNS neurotransmitter balance through the MGBA. As a potential endocrine organ, the gut microbiota produces various metabolites with signaling functions or hormonal properties, including neurotransmitters such as gamma-aminobutyric acid (GABA), dopamine, norepinephrine, and 5-hydroxytryptophan (Strandwitz et al., 2019; Cuesta et al., 2022; Liu et al., 2022; Omaliko et al., 2024). These signaling molecules mediate communication pathways through which the gut microbiota modulates the host’s neuroendocrine system, forming the MGBA (Wang et al., 2023; Teichman et al., 2020; Chen Q. et al., 2025).

Under hypoxic environments, the stability of these neurochemical pathways is readily compromised. In a mouse model combining hypoxic exposure with a high-fat diet, hypoxia significantly reduced the concentrations of 5-hydroxytryptamine (5-HT), norepinephrine (NE), dopamine (DA), and GABA in brain tissues, which occurred concurrently with alterations in gut microbiota diversity and anxiety- and depression-like behaviors (Qiao et al., 2025). Notably, the introduction of this high-fat diet as a concurrent dietary stressor further exacerbated these gut dysbiosis patterns and neurotransmitter imbalances, highlighting a potential synergistic interaction between metabolic stress and environmental hypoxia (Qiao et al., 2025).

At the neuroendocrine level, environmental hypoxia can disrupt the homeostatic feedback of the hypothalamic–pituitary–adrenal (HPA) axis, driving elevated glucocorticoid release that correlates with enhanced central neuroinflammation and synaptic damage (Gong et al., 2025). Gut microbiota-derived metabolites (e.g., via cytochrome P450/drug transporters) indirectly modulate brain function (Bai et al., 2022). Although a direct causal link establishing the gut microbiota as the obligate mediator of this hypoxic HPA activation remains to be fully elucidated, peripheral changes may interact with these neuroendocrine pathways. Together, these findings indicate that neuroendocrine cascades and altered neurotransmitter profiles represent plausible pathways through which high-altitude environments and concurrent gut dysbiosis may associate with neural dysfunction.

### 4.4 Interconnections among mechanisms and evidence transparency

The preceding sections follow a progressive logic: starting from gut microbial metabolic dysregulation, moving to gut-to-systemic transmission via humoral and neural routes, and culminating in brain-level consequences including oxidative stress and neuroendocrine imbalance. These layers are not independent; for example, barrier dysfunction can amplify systemic inflammation, which may further impair gut integrity and exacerbate central oxidative damage. However, direct evidence for causal interconnections in humans is limited. Most findings derive from rodent studies with heterogeneous protocols, and few investigations have simultaneously measured multiple layers within the same cohort. Thus, while the proposed cascade provides a coherent framework, its temporal and causal relationships require rigorous testing in longitudinal, multi-omics studies.

## 5 Microbiota-targeted neuroprotection via MGBA

In the high-altitude setting, microbiota-targeted neuroprotection can be defined as deliberate modulation of gut microbial ecology and/or microbial functional outputs with the goal of potentially reducing hypoxia-associated neural injury and preserving cognition through MGBA pathways. This framing goes beyond “dysbiosis descriptions” by emphasizing actionable intermediates: intestinal barrier integrity, systemic inflammatory tone/endotoxemia signals, microbial metabolites (e.g., short-chain fatty acids (SCFAs)), neurovascular/BBB stability, and glial activation state. Canonical MGBA models posit parallel communication channels—immune, metabolic, endocrine, and neural (vagal/enteric)—through which microbiota-driven shifts in barrier integrity and microbial metabolites can plausibly couple gut-state perturbations to CNS inflammation, neuroplasticity, and behavioral phenotypes (Margolis et al., 2021; Cryan et al., 2019). High altitude provides a particularly compelling testbed for microbiota-based neuroprotection because hypobaric hypoxia and related stressors can simultaneously perturb the microbiota, compromise gastrointestinal barrier function, and amplify inflammatory responses—all of which can converge on neuroinflammation and hippocampal-dependent cognitive outcomes (McKenna et al., 2022; Qi et al., 2025; Guo et al., 2025).

### 5.1 Mechanistic neuroprotective targets along the MGBA under hypoxia

#### 5.1.1 Barrier-first protection: intestinal barrier as the upstream gatekeeper, BBB as the downstream amplifier

Evidence consistently demonstrates that hypoxia, combined with physical exertion and dietary shifts, causes intestinal epithelial injury and increased permeability. This barrier failure facilitates the translocation of microbial products, such as LPS, into the circulation, thereby driving systemic inflammation, BBB stress, and downstream neuroinflammatory cascades. Intestinal injury under hypoxia and the role of reduced splanchnic perfusion (especially with exertion) are explicitly discussed as a driver of barrier dysfunction (McKenna et al., 2022).

Altitude-specific experimental evidence supports a microbiota-dependent barrier pathway. In a mouse high-altitude hypoxia model integrated with fecal microbiota transplantation (FMT), hypoxia reduced memory performance and was accompanied by microbiota shifts; importantly, FMT from hypoxia-exposed donors transmitted memory impairment along with increased intestinal and BBB permeability in recipients—strongly implicating the microbiota as an upstream driver of barrier-linked neurocognitive injury (Li et al., 2025). This altitude-specific chain is mechanistically consistent with canonical BBB biology: germ-free mice exhibit increased BBB permeability, and microbial colonization or microbial metabolites can influence tight-junction expression and BBB function (Braniste et al., 2014).

A key neuroprotective implication arises from the MGBA: interventions that maintain or restore gut barrier function (tight junctions, mucus layer, and epithelial repair) in hypoxic environments are likely to confer significant benefits to the brain. By curtailing the systemic inflammatory spillover that challenges the cerebral vasculature, these interventions can mitigate secondary stress on the neurovascular unit and BBB. Consequently, this reduces the likelihood of unchecked microglial activation and the associated vulnerability of the hippocampus to injury.

#### 5.1.2 Neuroimmune modulation: microglial set-point and inflammation transmission

Microglia act as a core effector layer for neuroinflammation-driven cognitive vulnerability. The host microbiota is required for normal microglial maturation and immune responsiveness, implying that microbiota modulation can shift baseline neuroimmune “set-points” (Erny et al., 2015). In high-altitude cognitive impairment paradigms, microglial activation is placed downstream of barrier dysfunction and systemic inflammatory spillover, linking gut events to hippocampal injury (Su et al., 2024). From a therapeutic standpoint, this framework suggests that microbiota-targeted interventions can protect the brain by either reducing peripheral inflammatory inputs to the CNS (often through restoring barrier integrity) or by recalibrating the immunometabolic signals that govern glial activation thresholds. In this context, it is methodologically advantageous to treat microglial activation as an intermediate endpoint—a quantifiable mechanistic bridge—rather than solely as a cognitive outcome, allowing for more precise tracking of how gut dysregulation translates into neurocognitive decline.

#### 5.1.3 Functional outputs: SCFAs, inflammatory mediators, and nutrient handling

Microbial metabolites provide a function-centric route to neuroprotection that is especially relevant when taxa changes are heterogeneous. SCFAs are frequently highlighted because they can modulate immune signaling and barrier integrity, including BBB-relevant pathways. In the high-altitude context, several lines of evidence make SCFAs particularly appealing: hypoxia and altitude diet shifts can change SCFA-producing communities; SCFAs are measurable biomarkers of functional microbiome output; and barrier outcomes (tight junctions, mucus production) are mechanistically linked to SCFA signaling (O’Riordan, 2022; Fock and Parnova, 2023).

At the same time, SCFAs may not always be protective. In non-altitude neurodegenerative models, microbiota-derived SCFAs can modulate microglia and promote amyloid plaque deposition, illustrating context dependence and the importance of disease stage and system state (Colombo et al., 2021). A mature perspective for high-altitude research recognizes that SCFAs are high-leverage mediators that often support barrier and immune homeostasis, but their net effect depends on host context; therefore altitude studies should report both metabolite shifts and mechanistic intermediates (permeability markers, cytokines, glial state) rather than inferring benefit from SCFA changes alone (Colombo et al., 2021; O’Riordan, 2022; Fock and Parnova, 2023).

A second functional axis emerging in high-altitude cognitive work is nutrient handling. A human-to-mechanism study in high-altitude migrants linked dysbiosis and barrier dysfunction to impaired nutrient absorption (including amino-acid handling), alongside inflammatory spillover and hippocampal injury; targeted microbial intervention restored barrier integrity and improved functional readouts relevant to neurocognitive outcomes (Su et al., 2024). This supports a broader neuroprotection model in which microbiota interventions may protect the brain not only by reducing inflammation, but also by restoring substrate availability relevant to neurotransmission and neurotrophic support.

#### 5.1.4 Human dynamics: time course, acclimatization, and phenotype stratification

Human data emphasize that gut microbiota changes with altitude are time-dependent and may track acclimatization phenotypes. A Genome Biology time-series analysis identified rapid microbiota responses to high-altitude hypoxia and implicated specific taxa (e.g., Blautia-related signals) in maintaining intestinal health and supporting adaptation (Ma et al., 2025). Longitudinal studies also report microbiome remodeling during acute and prolonged exposure in healthy adults (Karpęcka-Gałka et al., 2025), and expedition studies show that altitude exposure plus diet changes alter microbiome composition and functional pathways in mountaineers (Li et al., 2016). These findings matter for neuroprotection design because they argue for phase-specific interventions (early ascent vs. chronic residence) and for stratifying by baseline microbiome, diet, and exertion.

### 5.2 Intervention modalities and evidence for neuroprotection under high altitude

Conventional microbiota-targeted interventions, such as probiotics, prebiotics, and fecal microbiota transplantation, have been extensively studied in diverse clinical settings. Among these, emerging evidence supports their neuroprotective efficacy at high altitude, underscoring their promise as therapeutic strategies against hypoxia-induced brain injury. While these preclinical interventions show promise, their efficacy in humans remains to be validated.

#### 5.2.1 Probiotics and “altitude-adapted” strains

A particularly strong narrative for high-altitude neuroprotection is that altitude-associated microbial resources may contain strains with functional properties beneficial under hypoxia. In a chronic high-altitude exposure mouse model, *Lactobacillus johnsonii* HL79 (isolated from a healthy Tibetan child) improved working-memory performance and antioxidant capacity while modulating microbiota features—providing a direct example of probiotic-style neuroprotection through microbiota–gut–brain pathways (Zhao et al., 2025). In humans, the clinical literature is earlier and often focuses on acclimatization rather than cognition. A randomized study reported improved oxygen saturation and acclimatization metrics (including lower AMS scores) with bacteriotherapy/probiotic ingestion at high altitude (Yu et al., 2025). From a mature perspective, it is important to interpret current trials primarily as feasibility studies and probes of upstream physiological signals. While they provide valuable proof-of-concept for target engagement at the systemic level, future investigations must advance the field by incorporating robust neurocognitive endpoints alongside a suite of mechanistic biomarkers—including validated markers of intestinal permeability, inflammatory mediators, and metabolomic profiling. Such integration will be essential to establish definitive links between peripheral interventions and clinically meaningful brain outcomes.

#### 5.2.2 Prebiotics/dietary polysaccharides: scalable barrier–immune stabilization

Prebiotic-style interventions are attractive at altitude because they are scalable and can be incorporated into diet protocols. A recent study reported that chicory polysaccharide mitigated hypoxia-induced intestinal injury and cognitive deficits in mice, associated with gut microbiota remodeling and IL-6/IL-6R/STAT3 pathway inhibition (Shi et al., 2026). Notably, this study also highlights SCFA measurement as part of a mechanistic readout, consistent with function-centric neuroprotection logic (Shi et al., 2026; O’Riordan, 2022). The mature framing here is that prebiotics may work by reinforcing epithelial integrity, dampening inflammatory signaling, and shifting microbial functional output, rather than by “restoring a single normal microbiome state.”

#### 5.2.3 Microbiome restructuring and causal inference: FMT and antibiotic perturbation

FMT provides high causal leverage in altitude models. The hypoxia-FMT work discussed above indicates that dysbiotic microbiota can transmit memory impairment and barrier permeability phenotypes (Li et al., 2025). In addition, chronic hypoxia studies examining microbiota–brain correlations provide convergent evidence that microbiota states track cognitive outcomes under long-term exposure (Zeng et al., 2025). However, for a neuroprotection section, it is important to position FMT carefully: FMT has shown promise in preclinical and clinical studies for its ability to modulate microbiota composition and associated outcomes, such as intestinal barrier function and immune responses, but in high-altitude settings, it is currently best treated as a mechanistic probe and a potential future therapy only under controlled conditions, due to challenges in standardization, safety screening, and feasibility (Su et al., 2024; Li et al., 2025; Braniste et al., 2014).

### 5.3 Integrated framework: how microbiota-targeted interventions may confer neuroprotection at high altitude

Within the altitude-related neurocognitive vulnerability framework, hypoxia perturbs microbiota ecology and intestinal physiology; barrier failure increases inflammatory spillover; systemic inflammation stresses the BBB and primes microglia; and hippocampal networks become vulnerable, producing measurable cognitive impairment. Evidence supporting this integrated loop includes (i) transmissibility of memory and permeability phenotypes via FMT under high-altitude hypoxia (Li et al., 2025), (ii) human-to-mechanism chains linking dysbiosis to barrier dysfunction, LPS leakage, neuroinflammation, and hippocampal injury (Su et al., 2024), and (iii) canonical demonstration that microbial state can regulate BBB permeability (Braniste et al., 2014).

Neuroprotective interventions can therefore be rationally categorized by which pressure point(s) they target: (i) Barrier-first strategies (prebiotics, barrier-supportive probiotics, butyrate/postbiotics) aim to reduce inflammatory spillover and secondarily protect BBB and glial stability (Shi et al., 2026; Bakshi and Mishra, 2025). (ii) Inflammation-rebalancing strategies (probiotics with immunomodulatory effects; diet strategies that reduce inflammatory amplification) aim to lower systemic cytokine load and reduce microglial priming (Su et al., 2024; Zhao et al., 2025). (iii) Function-first strategies (metabolite/nutrient handling restoration) prioritize measurable functional outputs (SCFAs, amino-acid handling, permeability biomarkers) over “normalizing” the entire community, enabling iterative optimization and clearer dose–response mapping (Su et al., 2024; O’Riordan, 2022; Fock and Parnova, 2023; Bakshi and Mishra, 2025).

Not a single intervention class is proven, but the most defensible near-term neuroprotection hypothesis at altitude is barrier-centered: stabilizing the gut barrier under hypoxia reduces inflammatory transmission, limits BBB stress, and lowers the probability of maladaptive neuroinflammatory cascades—while function-first readouts such as SCFAs, intestinal fatty acid-binding protein (I-FABP)/zonulin and cytokines provide tractable biomarkers for mechanism-guided clinical translation (Shi et al., 2026; Bakshi and Mishra, 2025).

## 6 Challenges and future directions

Despite substantial progress in elucidating the role of the MGBA in high-altitude neuropathology, several critical challenges must be addressed to translate these findings into clinical applications.

### 6.1 Limitations of current research

#### 6.1.1 Causality vs. correlation

Foremost among these is the issue of causality: while associative studies have consistently identified alterations in gut microbial composition under high-altitude conditions, and FMT experiments in animal models have provided causal evidence linking specific microbial configurations to cognitive impairment, human studies remain largely correlational, leaving unresolved whether dysbiosis is a cause or consequence of host physiological changes. In particular, it remains unresolved whether the observed microbial shifts are primary pathogenic drivers or rather secondary adaptive responses to hypoxia-induced changes in intestinal physiology, diet, or host metabolism. Distinguishing between these possibilities is essential for the rational design of microbiota-targeted interventions. Furthermore, nearly all human evidence is correlational, not causal. Observed microbial shifts may reflect adaptive responses to hypoxia or inflammation, rather than primary drivers of brain injury, and reverse causality cannot be excluded.

#### 6.1.2 Heterogeneity and lack of large-scale longitudinal studies

This causal ambiguity is compounded by the scarcity of large-scale, multicenter, long-term clinical studies, as current evidence derives predominantly from small-sample observational studies or short-term animal experiments with considerable heterogeneity in study designs, altitude exposures, and outcome measures, limiting cross-study comparability and meta-analytic synthesis. Additionally, microbiome research faces well-documented reproducibility challenges: results are sensitive to sample handling, sequencing platforms, and bioinformatic pipelines.

#### 6.1.3 Individual variability and lack of stratification

Adding further complexity, individual variability remains poorly characterized—host factors including genetic background, age, sex, baseline microbiota composition, and pre-existing health conditions significantly modulate responses to high-altitude exposure, yet most studies fail to stratify by these variables, potentially masking subgroup-specific effects and limiting generalizability.

#### 6.1.4 Temporal disconnects: short-term dysbiosis vs. long-term deficits

Beyond these well-recognized limitations, a critical yet underexplored dimension concerns the temporal relationship between short-term gut microbiota alterations and potentially long-lasting neurological deficits. Our review indicates that certain microbial changes occur rapidly upon acute high-altitude exposure and may partially normalize during prolonged acclimatization or after return to low altitude. However, it remains entirely unknown whether a transient episode of dysbiosis is sufficient to induce persistent cognitive impairment, or whether continuous microbial perturbation is required to maintain the pathogenic cascade. This distinction is clinically vital: if transient dysbiosis seeds long-term pathology, even short-term travelers could carry unappreciated risks; conversely, if neural dysfunction reverses upon microbiota normalization, dynamic on-demand interventions would be justified.

A key conceptual gap is the distinction between taxonomic recovery and functional recovery. Current evidence suggests these may be uncoupled. For example, the abundance of butyrate-producing *Roseburia* may rebound upon return to low altitude, yet the overall capacity for SCFA biosynthesis, particularly cobalamin-dependent pathways driven by *Blautia A*, could remain suppressed for extended periods. This raises the hypothesis that functional resilience lags behind taxonomic normalization, creating a ‘silent window’ of persistent vulnerability to neuroinflammation even when diversity scores appear normal. Consequently, the critical window for intervention may be much narrower than appreciated, possibly within the first days to weeks of exposure, rather than anytime during or after high-altitude stay.

Furthermore, the role of gut microbiota in neurological impairment should be viewed through a nuanced causal lens: it can act as a cause, a consequence, or a disease-modifying factor. Resolving these possibilities requires human studies with dense time-series sampling paired with animal models that manipulate exposure duration and microbial reconstitution timing. Until such data emerge, we caution against overinterpreting cross-sectional associations as evidence for causality or for the efficacy of any single intervention window.

#### 6.1.5 Lack of standardized intervention protocols and safety data

This variability is further compounded by the lack of standardized intervention protocols; probiotic, prebiotic, and postbiotic strategies vary widely in strain selection, dosage, administration timing, and outcome assessment, hindering reproducibility and clinical translation, while the safety and efficacy of microbiota-targeted interventions in vulnerable populations at high altitude remain entirely unexplored.

#### 6.1.6 Summary of limitations

Addressing these interconnected challenges will require a coordinated effort combining mechanistic rigor, longitudinal human cohorts, and standardized methodologies to establish the causal role of gut microbiota in high-altitude neuropathology and to develop evidence-based, personalized therapeutic strategies.

### 6.2 Future research directions

Future research must move beyond correlational observations toward mechanistic causality by employing integrated multi-omics approaches—combining metagenomics, metabolomics, transcriptomics, and proteomics—to comprehensively characterize host-microbiota interactions and identify key functional bacteria and effector molecules that mediate neuroprotective effects. These systems-level analyses should be complemented by deep mechanistic investigations that elucidate precise strain-metabolite-receptor pathways, mapping how specific microbial metabolic capacities engage cognate host receptors and downstream signaling cascades in intestinal epithelial cells, immune cells, and neurons, with organoid and microphysiological system models offering tractable platforms for such dissection.

A notable gap in the current literature is the systematic underreporting of sex-dependent effects. Given that sex hormones modulate both gut microbiota composition and the host’s response to hypoxia, future studies should be explicitly designed to test for sex differences in MGBA-mediated neurocognitive outcomes under high-altitude exposure. This includes the use of both sexes in animal experiments, sex-stratified analyses in human cohorts, and, where possible, investigation of hormonal influences on acclimatization and cognitive resilience.

Notably, clinical intervention studies remain scarce, underscoring the urgent need for well-designed human trials to validate the efficacy of MGBA-targeted strategies. Furthermore, given that the microbiota functions as an integrated network, future research should not anchor exclusively on single strains but rather adopt a holistic concept that considers the collective role of the microbial community as a functional unit, with attention to synergistic and competitive interactions among taxa and their combined impact on host health.

Given the time-dependent remodeling of gut microbiota under acute vs. chronic exposure, future studies must also map the spatiotemporal dynamics of MGBA changes across different altitudes and exposure durations to identify critical windows for phase-specific interventions, determining whether early probiotic supplementation prevents the initial dysbiotic cascade or whether late-stage interventions can reverse established impairments. Equally important is exploring the underexamined bidirectional regulation of the axis, investigating how central nervous system stressors modulate gut microbial ecology via autonomic nervous system output and neuroendocrine signaling to complete our understanding of gut-brain crosstalk.

Ultimately, clinical translation will require the development of microbiota-based biomarkers for predicting individual acclimatization capacity and susceptibility to cognitive impairment, leveraging machine learning models that integrate baseline microbiota features, host genetics, and clinical parameters for risk stratification and personalized prevention, alongside randomized controlled trials with robust neurocognitive endpoints and validated biomarkers of intestinal permeability and systemic inflammation to establish efficacy in humans. Future studies should prospectively validate whether candidate biomarkers such as LPS, I-FABP, IL-6, and *Blautia A* can effectively stratify high-altitude cognitive risk, as conceptualized in Figure 1.

### 6.3 Translational roadmap for microbiota-targeted interventions

To bridge the gap between preclinical promise and real-world application, a structured translational framework is proposed for microbiota-targeted interventions in high-altitude populations, addressing feasibility, safety, patient selection, and regulatory challenges.

#### 6.3.1 Clinical feasibility and practical implementation

High-altitude settings demand simple, scalable, and low-resource interventions. Probiotics/prebiotics are preferred for routine use: they are oral, shelf-stable, low-cost, and easy to distribute in remote plateau regions, suitable for both short-term travelers and long-term residents. FMT, while mechanistically powerful, is reserved for severe cognitive impairment or refractory cases due to logistical complexity. Interventions should be timing-stratified: pre-ascent prophylaxis for acute mountain sickness prevention, and maintenance supplementation during prolonged residence to sustain gut-brain homeostasis.

#### 6.3.2 Safety and adverse effect considerations

Probiotics and prebiotics are generally safe in healthy populations, with mild, transient, and self-limiting gastrointestinal symptoms such as bloating or diarrhea being the most common adverse events; however, they are contraindicated in immunocompromised individuals due to potential risks of bacteremia. In contrast, FMT carries notable safety concerns, including the risk of pathogen transmission, gastrointestinal disturbances, and rare immune reactions, requiring rigorous donor screening for infectious agents and healthy gut microbiota profiles, as well as standardized preparation and administration protocols. Long-term safety data specific to high-altitude populations remain limited, necessitating close clinical monitoring during implementation.

#### 6.3.3 Patient selection and stratification

Microbiota-targeted interventions should be personalized based on individual characteristics and exposure profiles. Stratification may consider exposure type, distinguishing between short-term travelers and long-term residents, as well as baseline risk factors including pre-existing gastrointestinal conditions, older age, or a history of high-altitude cognitive impairment. Integration of microbiome biomarkers, such as reduced short-chain fatty acid production or dysbiotic signatures, can further enable identification of high-risk individuals for targeted preventive interventions.

#### 6.3.4 Regulatory and implementation challenges

Microbiota interventions face global regulatory gaps: probiotics are classified as dietary supplements (loose oversight), while FMT is regulated as a biological product (strict approval). Standardization of strain quality, dosage, and manufacturing protocols is critical for cross-study reproducibility. Local capacity building—including on-site microbiome testing and clinician training—is essential for sustainable deployment in high-altitude regions.

#### 6.3.5 Summary of translational priorities

The near-term focus is prebiotic/probiotic-based prevention for broad populations, with FMT reserved for severe cases. Key priorities include validating microbiome-based risk biomarkers, conducting multicenter RCTs in high-altitude cohorts, and establishing region-specific safety guidelines to ensure equitable and safe translation.

## 7 Conclusion

High-altitude hypobaric hypoxia is associated with disruption of gut microbial homeostasis, and it has been hypothesized that this may trigger a cascade of events along the MGBA that could contribute to neural dysfunction. Current evidence suggests that the dysbiotic state, characterized by reduced beneficial bacteria, intestinal barrier changes, and metabolic slterations, may be linked to interconnected mechanisms including LPS/TLR4-mediated neuroinflammation, SCFA deficiency, oxidative stress, and neurotransmitter imbalance, all of which have been proposed to converge on hippocampal plasticity and cognitive function. However, causality has not been firmly established in humans, and microbiota changes could also represent adaptive responses.

Critically, the gut microbiota emerges as a targetable mediator of high-altitude neuropathology. Preclinical evidence demonstrates that probiotic, prebiotic, and postbiotic interventions confer neuroprotection by restoring barrier integrity, dampening inflammatory signaling, and rebalancing metabolic outputs, though human data are still lacking. Integration of microbiota modulation into the high-altitude medicine framework requires a paradigm shift toward mechanism-guided, personalized interventions that consider exposure duration, individual baselines, and phase-specific therapeutic windows. Translating these fundamental insights into clinical strategies holds promise for preserving neural function in the growing populations residing in, traveling to, or working in high-altitude regions worldwide. However, a critical unresolved question is whether short-term, transient gut dysbiosis at high altitude can instigate long-lasting neurological deficits independent of ongoing microbial perturbation. Resolving this will require a paradigm shift from cross-sectional correlations to temporally-resolved, mechanism-driven studies that distinguish taxonomic from functional recovery, identify critical windows of dysbiosis-induced vulnerability, and establish whether microbiota changes serve as a cause, consequence, or modifier of neurological impairment. Addressing these questions will be essential to determine if, when, and for whom microbiota-targeted neuroprotection should be deployed.
